# Dementia and Video Games: Systematic and Bibliographic Review

**DOI:** 10.3390/healthcare12222310

**Published:** 2024-11-19

**Authors:** Martin Eckert, Thomas Ostermann, Jan P. Ehlers, Gregor Hohenberg

**Affiliations:** 1 Stabsstelle für Digitalisierung und Wissensmanagement, Hochschule Hamm-Lippstadt, 59063 Hamm, Germany; gregor.hohenberg@hshl.de; 2 Fakultät für Gesundheit, Universität Witten-Herdecke, 58455 Witten, Germany; thomas.ostermann@uni-wh.de (T.O.); jan.ehlers@uni-wh.de (J.P.E.)

**Keywords:** medicine, cognitive impairment, dementia, alzheimer’s disease, video games, serious games, exergames, systematic review

## Abstract

Background/Objectives: This systematic and bibliographic review examines publications in the field of dementia and video game research from 2004 to 2023. The main objective is to assess developments and trends in video game technology for dementia care and detection. Methods: The PubMed database was the primary source for publications. PRISMA guidelines were applied to structure this review. Ten variables were defined, investigated, and split into three main categories: bibliographic, medical, and technical. Results: The results were synthesized using a quantitative approach to reduce bias through interpretation. Of 209 initial results, 77 publications have been included in the investigation. More studies focus on rehabilitation over assessment and detection of dementia. Clinical trials are typically conducted with limited participants. The most populated trials rarely enrol over 300 subjects. On average, around 38 subjects were enrolled in the trials. These studies are commonly supervised by a trainer or technology specialist, suggesting a technology gap in familiarity in the trial demographic. Conclusions: Most interventions assessed were custom-designed applications with a specific outcome, focusing on physical activity and cognitive exercises. As the first of its kind, this publication focuses on the technical aspects of applied technologies and development methods. Using video games to treat and detect patients with cognitive impairments like dementia can benefit healthcare professionals, caretakers, and patients.

## 1. Introduction

Neurodegenerative diseases are a growing global problem [1]. Many Western societies face an expanding, aging population. Most forms of neurodegeneration take a strain on the life quality of patients and have an impact on overall mortality [2]. The expansion in dementia-related disorders, such as Alzheimer’s or Parkinson’s disease, makes it essential to find innovative approaches to detect, treat, and understand these kinds of diseases.

Dementia treatment is based on cognitive stimulation to prevent the rapid decline of neurocognitive functions. The argument is that using video games for cognitive stimulation in dementia patients, specifically in preventing dementia, is beneficial. This research project addresses this point precisely and provides an overview of the current research and its technology.

### 1.1. Background

The effect dementia can have on individuals, families, and health systems cannot be overstated. Dementia is characterized by a progressive decline of cognitive abilities, memory loss, impaired thinking, and behavioral changes, as found by Roberts and Knopman [3]. With an estimated 50 million people worldwide diagnosed only with dementia and 10 million new cases every year, this figure is set to triple by 2050. The number of people with dementia is going to reach 82 million in 2030 and a projected 152 million in 2050. Dementia is a global public health priority [4].

The German medical guideline recommends medication, regular exercise, cognitive stimulation, and a healthy diet as treatment. Furthermore, the 2017 German medical S3 guideline states that a supportive environment and social interaction are essential; see [5] for reference. Regular exercise and cognitive stimulation can be addressed, especially using modern entertainment technologies such as video games. Video games are defined as interactive electronic experiences. They can influence behavior, cognition, and emotions alike. Research explores topics such as attention and problem-solving, brought up by Rodrigo-Aynguas et al. [6] and emotional well-being, as discussed in Pallavicini et al. [7].

Once a game is applied to a context other than entertainment, it is referred to as a serious game and is often used in healthcare or education. If a video game is taken from a cognitive domain to a physical domain, it is called an exergame—a combination of exercise and game coined in the early 2000s.

Multiple studies explored the benefits of video games on cognitive stimulation and physical rehabilitation for dementia patients. However, there is a lack of comprehensive analysis focusing on the technical aspects of these interventions. Specifically, the identifiable literature does not sufficiently cover the types of technologies and methods used to create the intervention. Without this understanding of the technological implementations, opportunities for further research and improvement of said technologies may be missed or duplicated.

To address the research gap, this systematic and bibliographic review examines publications in dementia and video game research from 2004 to 2023. The main objective is to assess developments in video game technology for dementia care and detection.

Focusing on technical aspects such as output devices, add-on technologies, autonomy in usage, and development regimes. This review contributes to science by offering insights that can guide future projects and inform the design of more effective interventions.

### 1.2. Overview

Early research in that field can be dated back to the year 2004, conducted by Jimison et al. [8]. The following paragraph summarizes recent reviews in the fields of serious gaming, exergaming, augmented reality (AR), and virtual reality (VR) in combination with dementia.

A 2023 review study by Zhu et al. [9] examines mobile apps for people with dementia, aiming to identify designs and evaluations. The review looked at 20 publications with the overall finding that serious games can improve cognitive skills, help with therapy, and musical apps can help slow memory loss. Another study finding is that personal life apps are effective in improving independent living for people with dementia.

The review of Saragih et al. [10] from 2022 focuses on clinical trials to assess the overall effects of serious games on people with dementia. Twelve studies found that serious games can have an overall positive impact on the patient’s cognition. There is an additional finding that serious games have a positive influence on depression. Results conclude with the potential of using serious games in dementia care but also mention the need for future research to learn about long-term effects.

In a recent system review from 2023, Cai et al. [11] are writing about the impact of exergaming on people with mild cognitive impairment (MCI) and dementia. The review analyzed ten randomized controlled trials and found that exergaming significantly positively affected cognitive function and physical performance. They did, however, not show significant improvements in activities of daily living or quality of life. The authors highlight that the findings can only be interpreted with caution because of the heterogeneity of the clinical trials. The full benefits of exergaming should be examined in further research.

Hill et al.’s systematic review from 2017 [12] examined the effects of computerized cognitive training (CCT) on older adults with mild cognitive impairment (MCI) and dementia. During the review, 29 randomized controlled trials were identified and analyzed. Findings suggest moderate improvement in global cognition and psycho-social functioning for MCI patients. Strong effects on patients with dementia were only observed in trials applying immersive technologies such as virtual reality. The authors conclude that more extensive trials need to be conducted to explore the long-term efficacy of CCT, especially in a virtual reality environment.

In 2022, Flynn et al. [13] looked at key stakeholders in using AR and VR solutions for older people with dementia. They took a qualitative approach, collected experiences and perceptions of key stakeholders involved in the care, and used the solutions.

The review included 14 studies that revealed three analytical themes: entering virtuality, a virtual world, and returning to reality.

Findings suggested that VR can provide positive experiences for patients with mild cognitive impairment (MCI) and have a real-life impact. They also emphasize the importance of designing VR interventions with great sensitivity and involving the key audience in the design process to ensure the use of helpful technology in dementia care.

In summary, the most recent research often finds positive outcomes regarding experience, cognitive function, and physical performance. It is also concluded that more research is required to establish the fields of exergaming and serious gaming in the treatment of patients with dementia. This paper analyzes research conducted on dementia and video games over the last twenty years. It provides a comprehensive overview, focusing on technical aspects of applied technology and development methods. It also may help researchers make informed decisions in future projects in this field.

## 2. Materials and Methods

This section describes the method applied to the review. The research method was based on the preferred reporting items for systematic reviews and meta-analyses (PRISMA) introduced by Moher et al. [14].

### 2.1. Search Strategy and Identification of Articles

The primary source of publications for this review is the PubMed database, hosted by the National Library of Medicine in the United States. The initial query for relevant publications was designed based on the research topic of ’Dementia and Video Games.’ This preliminary query, including Alzheimer’s disease as the most common form of dementia, aimed to capture a broad initial spectrum of publications related to this topic; see the Risk reduction of cognitive decline and dementia: WHO guidelines [4]. The initial query was translated and refined using PubMed’s advanced search capabilities to enhance its precision and specificity. The initial query can be reviewed in Listing 1.

**Listing 1.** Initial search query.((Dementia) OR (alzheimers disease)) AND ((video games) OR (computer games) OR (exergames) OR (serious games))

It involved utilizing Boolean operators and applying Medical Subject Headings (MeSH) to find additional publications to address the intersection of dementia and video games specifically. The expanded query is attached in the Appendix B: Extended Search Query; see Appendix B.

### 2.2. Article Screening and Eligibility

Early on, during the screening of the title, abstract, and keywords, a set of false-positive keywords was identified and transferred into a list of exclusion terms. This enabled the functionality to exclude publications with identified terms from the set automatically. The function has been applied to the title and keywords of the results. The list of false-positive words is included and can be found in Appendix C: Exclusion Terms.

The articles and meta-data retrieved from the initial search query were managed using Zotero 7, an open-source reference management software. Zotero enabled an efficient organization, screening, and annotation workflow during the entire process. After automatic exclusion, the manual process of screening titles, abstracts, and keywords took place. The publications were excluded from the result set if they did not align with the inclusion and exclusion criteria. Review articles were not included in this review because of a lack of comparability and different review approaches. A selection of reviews has been included in Section 1.2. The inclusion and exclusion criteria have been defined for the manual screening process. An overview of the inclusion and exclusion criteria can be seen in Table 1.

**Table 1:** Inclusion and exclusion criteria for the screening process.

Criteria Type	Description
**Exclusion Criteria**	No valid DOI was provided or identified through further research. Review articles due to lack of comparability and varying review methodologies. Publication is not available in the English language. Full-text is not in the open-access domain. Publications containing specific false-positive keywords identified during the initial screening.
**Inclusion Criteria**	Subjects and patients must have a neurological impairment (e.g., dementia, MCI, Alzheimer’s). Has been conducted as some form of clinical trial or study. See Table 2. Involves some form of video gaming, exergaming, or gamification. See Table 3.

**Table 2:** Medical variables and descriptions.

Variable	Description
**Intended Usage Scenario**	Distinguishes the proposed solution regarding its intended usage scenario.
*Detection and Assessment*	Focuses on an application to detect and assess the state of neurodegenerative disease.
*Rehabilitation*	Focuses on rehabilitation, care, and improvement of life quality.
**Advancement of Clinical Research**	Looks into the type of research that is conducted and referred to in the assessed publication.
*Single Case Study*	An examination of an individual case is used to gain in-depth insights into a particular observation.
*Pilot Study*	A small-scale study was conducted before a full-scale trial to evaluate key factors such as usability, time, cost, and expected risks. Pilot studies do not evaluate treatment effects.
*Feasibility Study*	Specific investigations into the conductibility and viability of a clinical trial. Feasibility trials feature a control group and a randomization group. The number of participants is lower than in a randomized clinical trial but higher than in a pilot.
*Randomized Clinical Trial*	Subjects are randomly assigned to intervention groups, evaluating the effect of the new treatment.
**Participation in Clinical Trials**	Numeric value, representing the subjects included in a clinical trial or study.
**Engagement Intensity with application**	A set of numeric values representing the engagement intensity of applied interventions. If the application solves a detection or assessment task, it is often conducted as a single intervention. Therefore, only a single application time is extracted from the publication.
*Overall time span*	Overall period of engagement with the application, measured in minutes.
*Number of weekly sessions administered*	Count of weekly sessions.
*Average duration of a single session*	Duration count in minutes
**Focus of Application**	Main focus area of the application.
*Motor*	Focuses on improving the motoric skills of the subject.
*Cognitive*	Focuses on improving cognitive skills.
*Mixed*	Combines cognitive and motoric skills.

**Table 3:** Variables regarding technical devices and development style.

Variable	Description
**Output Device**	Refers to the main technical output device of the application.
*Monitor*	A display attached to a computer to represent information.
*Monitor (touch)*	The touchscreen monitor allows interaction by touching it.
*Tablet*	Portable touchscreen device allowing interaction.
*Smartphone*	Handheld mobile device combining a phone, computer, and touchscreen.
*VR Headset*	Virtual reality headset that immerses the user in a 3D environment.
**Add-on Device**	Describes further technical additions used to enhance the gaming solution.
*Stationary Bike*	Fitness bike for active interaction with the digital environment.
*Kinect*	Infrared-based motion-tracking device for interaction with the digital environment.
*Sensors*	Various types of sensors, not specified.
*Physiomat*	Fitness mat triggered by foot to encourage exercise and interaction.
*Leap Motion*	Hand-tracking device to enable gesture control and interaction with a computer.
*Wii Remote*	Handheld device to track motions and interact with digital spaces.
*EEG*	Medical sensor that measures electrical brain activity used as an input controller.
*Wearable Sensors*	Sensors worn by the user during the application use.
*Balance Platform*	Step-on training platform to encourage stability and balance.
*Exerboard*	Specific rehabilitation device to enhance balance and coordination.
*Greenscreen*	Film-trick to replace the background with computer-generated graphics.
*Joystick*	Input device for computers, offering a simpler input method than a keyboard.
*Treadmill*	Fitness device to promote stationary walking and running exercises.
*Gamepad*	Handheld controller, often used with computers or gaming consoles.
*Hand Motion Tracker*	Device to track user’s hand and finger gestures.
*Dance Mat*	Step-on controller used in dance and rhythm games.
**Autonomy and Guidance**	Represents the state of autonomy and supervision during the usage.
*Supervised*	The subject has been supervised during the usage of the application.
*Unsupervised*	The subject is going through an unsupervised activity.
**Development Style**	Distinguishes the approach taken in software development.
*Designed*	Hand-tailored design approach to a specific use-case.
*Purpose Shift*	Use of off-the-shelf software where commercial software is repurposed.

The eligibility criteria are applied manually. Publications not matching the criteria are excluded. A single screener conducted the screening phase. Debatable publications were discussed in a meeting with all authors until an agreement was reached. After screening the articles, a set of variables worth extracting has been defined, and they are described in the following three sections.

### 2.3. Bibliographic Variables

Bibliographic variables aid as essential information about publication context and scientific impact. For a detailed overview of the publications and information about included publications with full titles, please refer to Appendix A. Two bibliographic variables, the year of publication and the country, were found to be relevant to deeper investigation and data extraction, see Table 4.

### 2.4. Medical Variables

For the medical context, variables such as the intended usage scenario, details of the conduct of clinical trials, and the level of engagement intensity are used to gain insight into the actual state of research. In the specific context of this publication, an application is defined as a combination of hardware and software that generates a solution for detection or rehabilitation purposes in the context of dementia.

### 2.5. Technical Variables

The technical variables have been defined to gain engineering insight into the development and creation of the applications. The following table shows variables and their subsets with a short explanatory text; see Table 3.

All variables have been manually extracted from the full text of the publication result set when possible. If a value was not available in the publication’s full text, the value and the publication have been excluded from the corresponding variable but not from the entire dataset. The following visualization techniques have been applied to better understand the collected data and variables. To visualize more complex datasets, we visualized them using a combination of violin and scatter plots. The relation between the device add-ons and their categories is realized using a Sankey plot. Figures have been generated using Python 3.10 and the following libraries for data mining and visualization: Pandas 2.1.4, Numpy 1.24.3, Matplotlib 3.7.1, Seaborn 0.12.2, and Plotly 5.9.0.

## 3. Results

The following sections show the extracted results. Simple data are brought into context by using tables, where more complex data are visualized.

### 3.1. Search Strategy and Screening

The search was conducted on Wednesday, 2 August 2023, and returned 209 initial results. The search was automated using the Python 3.10 library PyMed 0.8.9, which was used to interact with the PubMed API. The search output was parsed into a comma-separated file to increase readability. DOIs have been extracted into a single text file and imported into the Zotero 7 library management software. Zotero 7 was used to obtain clean meta-data and PMIDs when available. Furthermore, it served as a viewer to access and annotate publications in the screening and extraction phase.

**Figure 1:**
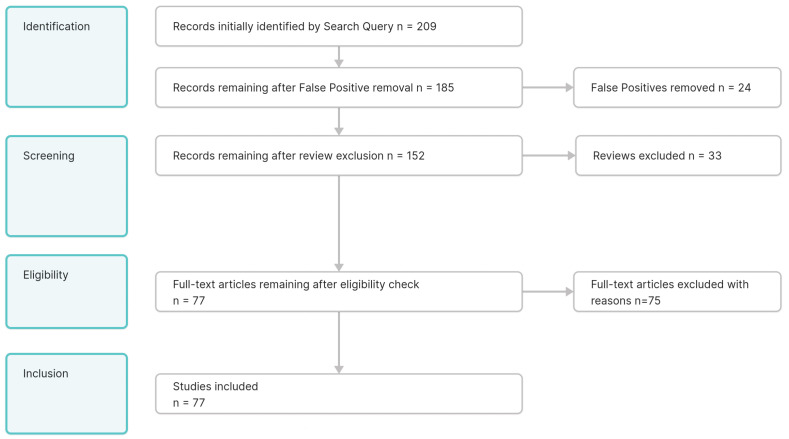
PRISMA Workflow. Visual representation of conducted stages, visualizing the individual steps and numbers of excluded publications in each phase.

The screening strategy was split into four phases. Phase One, Identification: Of the 209 initial publications, 24 false positives have been removed. Keywords in the title or abstract identified them. The negative list of exclusion keywords is part of the multimedia Appendix C. Phase Two screening: 33 reviews have been excluded in the second step after the false positives were removed. They were identified by reviewing the title and abstract. Phase Three eligibility: Of the remaining articles, 75 have been excluded after manual screening, based on inclusion and exclusion criteria defined in Table 1. Of the initial 209 publications, 77 remained to be included in this review. A table with all 77 included publications, including their references, can be found in Appendix A. Phase Four extraction of data are described in the next section of the paper. A visual overview of the applied process is presented in Figure 1 PRISMA Workflow.

### 3.2. Data Extraction and Presentation

The data have been manually extracted from the full text and transferred to a result table for further investigation. If any extracted data point was not clear, this data point was marked and discussed in a forum of all authors until consent was reached. The collected data were cleaned and unified using the open-source software Open Refine 3.5.2 to correct typos or mistakes during data entry. Cleaned results were parsed into a comma-separated file.

### 3.3. Bibliographic Results

This section contains the results of the bibliographic analysis, the timeline investigation, and the countries of origin. The results of publications returned by the initial search allowed the removal of a time limit for the research scope.

#### 3.3.1. Timeline of Publications

**Figure 2:**
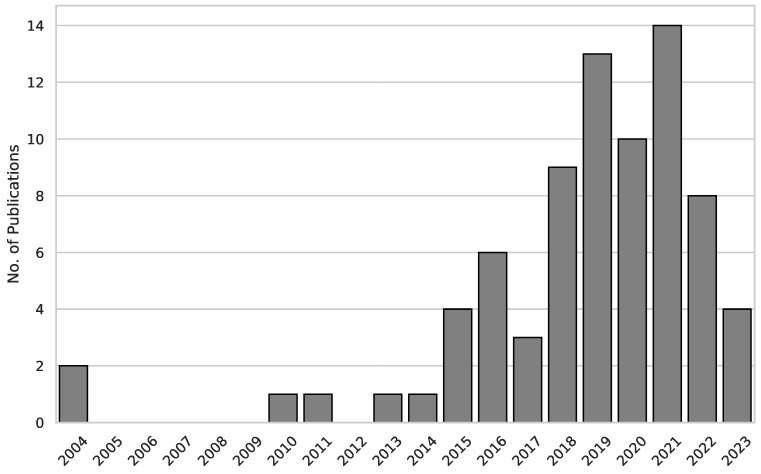
Timeline of publications. The bars indicate the number of publications for a specific year. The years range from 2004 to 2023. The number of publications varies from a minimum of 0 to a maximum of 14 in 2021.

Jimison et al. assessed the use of computer technologies to detect cognitive status in elders back in 2004 [8]. After that, it became quiet for some years, but in 2013, Kloos et al. started using sensor-based maps in the therapy of elderly patients [15]. In 2015, we saw a growing number of research activities, reaching their peak in 2021, followed by a decline in publication in recent years. A visualization of the publication timeline can be seen in Figure 2’s timeline of publications.

#### 3.3.2. Country of Origin

In total, 14 of the 77 publications were conducted in the United States of America, followed by the Netherlands, which had eight publications. Canada and France split the third place with seven publications over the time frame (2004–2023). The entire dataset is viewable in Table 5’s publications by country.

### 3.4. Medical Results

This section describes the findings of the medical scope of this review paper. Data from the included publications regarding the intended usage scenario, maturity of clinical research, and participation numbers in clinical trials have been extracted. Furthermore, the medical results include an analysis of an applied dose of the application applied in clinical trials.

#### 3.4.1. Intended Usage Scenario

The usage scenario describes whether the application detects or assists in rehabilitation scenarios. The findings include 76 publications. A rough quarter of publications (n = 21) are used to detect and assess neurodegenerative diseases. The largest share of publications (n = 55) is dedicated to the rehabilitation scenario and improving life quality.

#### 3.4.2. Advancement of Clinical Research

The clinical research stage is an aspect to consider regarding the maturity of the proposed solutions. The publications are classified as single case studies, feasibility studies, pilot studies, and clinical trials; see Table 6. One publication was removed due to this variable’s lack of extractable information.

Combined with Section 3.4.3, this metric provides a comprehensive understanding of the state of clinical research in the detection and care of people with dementia.

#### 3.4.3. Participation in Clinical Trials

**Figure 3:**
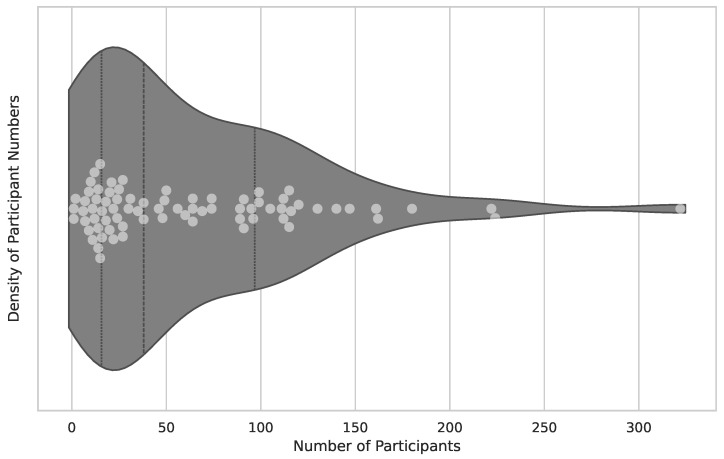
Participation in clinical trials. The figure combines the visualization of the subjects’ distribution in the clinical trials. It is a combination of a violin and a scatter plot to provide insight into the actual dataset. The x-axis represents the number of participants. Overall publications, excluding non-existent information, are 76 publications in total. The median of included subjects is 38, the lower quartile is 16 subjects, and the upper quartile is 97 subjects. Several outliers with higher numbers can be detected with 322 subjects included [16].

The number of subjects in a clinical trial is vital to its scientific significance for several reasons (sample size, representation, diversity, reliability). Therefore, the publications regarding subjects included in the clinical trial have been analyzed, and results are visualized in Figure 3.

#### 3.4.4. Engagement Intensity

**Figure 4:**
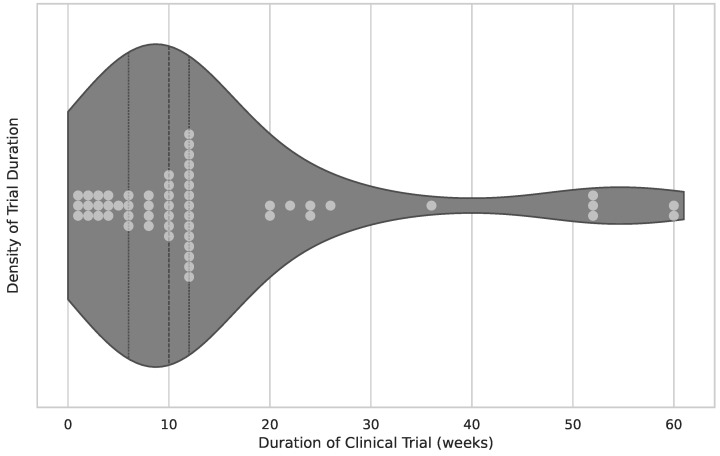
Overall duration of study/trial. The figure combines the visualization of the overall timespan the gaming application was used by a subject (in weeks). The x-axis represents the number of weeks. Fifty-five publications provided data. The median of included subjects is ten weeks, the lower quartile is six weeks, and the upper quartile is 12 weeks. Some outliers can be observed on the right-hand side.

**Figure 5:**
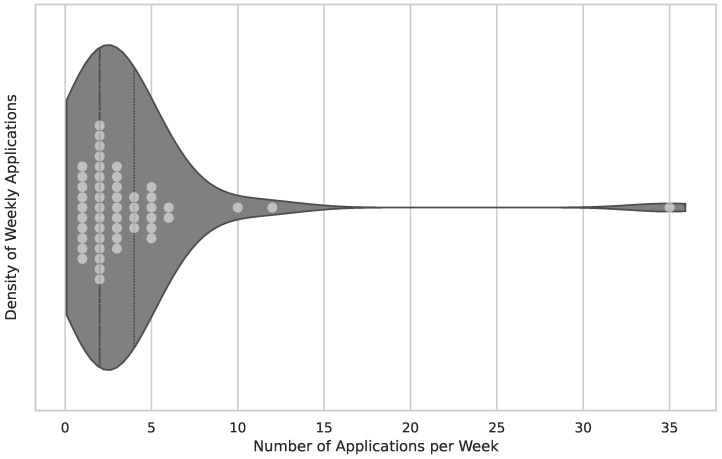
Number of weekly sessions. The figure is the visualization of the number of application treatments within a week. The x-axis represents the number of application treatments. Fifty publications provided the data. The median of application treatments given to a subject is two times per week. Also, the lower quartile is two times per week, and the upper quartile is four times per week. A very far outlier can be observed on the right-hand side at 35 times per week, as described by Wu et al. [17].

**Figure 6:**
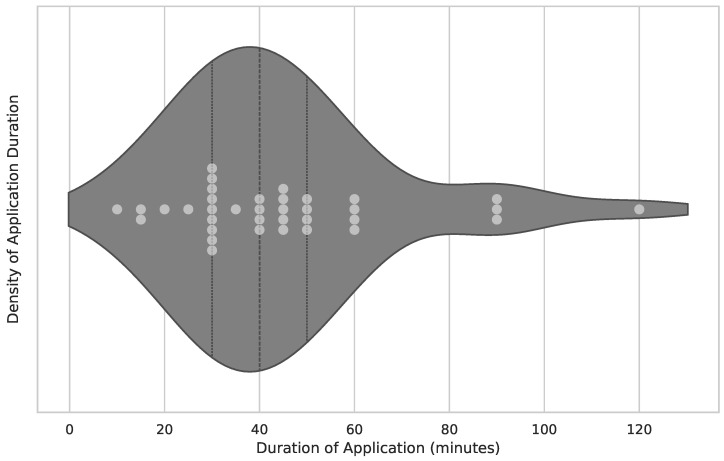
Single session duration. The figure combines the visualization of the actual session length of a single session. The x-axis represents the number of minutes spent during the session. Thirty-six publications provided the data. The median of the session length is 40 min. The lower quartile is 30 min, and the upper quartile is 50 min. The shortest session is held by Werner et al. (10 min) [18] and the longest session by Fasilis et al. (120 min) [19].

In this section, the results of the application intensity usage are presented. The intensity is depicted using a temporal approach. There are three stages in which the engagement intensity is measured:

1. Overall time span of engagement with the application, as depicted in Figure 4’s overall duration of study/trial.
2. Number of weekly sessions administered, as shown in Figure 5’s number of weekly sessions.
3. The average duration of a single session, measured in minutes and visualized in Figure 6.

It is important to note the overall size of the dataset for each category. Multiple publications did not contain the required information on doses.

The average clinical trial had 38 subjects and followed a protocol with a duration of 10 weeks of application usage. Sessions were administered twice weekly and lasted 40 min each.

### 3.5. Technical Results

This section describes findings of the technical scope. Data from the included publications regarding output devices, add-on devices, autonomy, and development have been extracted.

#### 3.5.1. Output Device

The proposed solutions differ in the output device the developer chooses. The Table 7 Output Device shows that the most common output devices are regular computer monitors (n = 30), followed by tablets with touchscreen functionality (n = 20). Nine publications feature a VR headset. Further findings included six publications with unknown output devices, four outliers, two monitors with touchscreen functionality, and two smartphones. The publications without extracted information for output devices have not been listed in Table 7.

#### 3.5.2. Add-On Device

**Figure 7:**
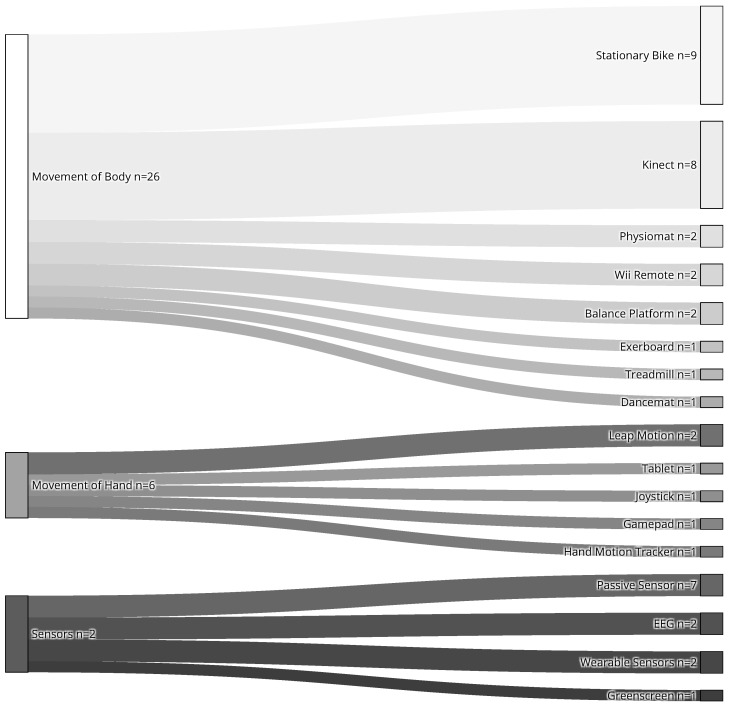
Add-on devices. The Sankey plot illustrates the device addons with their three broad categories (‘Movement of Body’, ‘Movement of Hand’, and ‘Sensors’.) The right side of the plot contains the specific subcategories of applied addons. The total number of collected datasets is 34.

This section explores the integration of various add-on devices. Additional devices can play a crucial role in designing the exergaming experience for the subject and individuals with dementia. The used devices have been categorized by the type of external input they process. When the entire body is required to use the add-on device, the category is ’Movement of Body’. If only hands and gestures are used to input, the category is ’Movment of Hand’. Some publications do not name the device, or it does not match any of the above categories. Those devices have been classified as ’Sensors’. The distribution of the individual devices into the categories is visualized using a Sankey plot in Figure 7’s add-on devices.

#### 3.5.3. Autonomy and Guidance

This section covers the aspects of supervision and autonomy while using various solutions. Supervised solutions for exergaming benefit from immediate assistance to ensure safe and ideal participation. Progress can be assessed in real-time, and structured interventions can be applied. Furthermore, social interaction with supervisors is made more accessible. The unsupervised approach nurtures independent interaction without constant guidance and the flexibility to choose one’s activity and intensity levels as a subject. Also, another benefit is the lower staff requirements and cost reduction. The numbers are available in Table 8.

#### 3.5.4. Development Regime

One technical aspect in developing and using applications for people with dementia is the development regime. The term refers to the approach taken in producing the software: whether it was meticulously designed for the intended purpose or if a shift in purpose was applied to consumer-grade software, as listed in Table 9.

Fifty publications were specifically designed for dementia to match patients’ requirements. Only fifteen publications repurposed existing off-the-shelf software, and twelve publications did not make any statements on the software-development process.

## 4. Discussion

The results underline interactive technologies’ positive role in detecting and managing cognitive impairments. The review started to collect the momentary state of research in the cross-over field of computer games and patients with cognitive impairment, specifically dementia and Alzheimer’s disease. In the last twenty years, 77 projects were identified to match the inclusion criteria. Clinical trials have been conducted, with a median of 38 participants. The majority of studies used regular computer monitors as a primary output device. Stationary bicycles were the most commonly used add-on technology. Proposed applications were often developed for specific use cases rather than repurposed from off-the-shelf software.

### 4.1. Principal Findings

The first finding is that the examined publications focus on rehabilitation trials over early detection and diagnostic assessment of mild cognitive impairment (MCI) and dementia. Detection has been researched and is a well-working concept. Therefore, the focus is rehabilitation solutions and improving patients’ quality of life. The conducted studies have limited sample sizes. Thirty-eight participants with outliers in both directions are the average clinical trial participants in this field of research. The low numbers may keep the healthcare sector from concluding and including those technologies in regular care. To assess this topic, there is a need for larger and more diverse studies to provide stronger evidence and integrate video game technology into routine dementia care.

In the next finding, widely adapted technologies are used in most trials. Standard output devices such as computer monitors and tablets indicate a preference for familiarity. The same preference is observed for device add-ons. The most used add-on device is the stationary bicycle, which is easy to use and does not require extensive explanation before use.

Most interventions were found to be conducted under professional supervision, suggesting potential usability problems that might hinder adoption in regular care settings. An explanation is that the complexity and novelty of modern technologies contribute to patients’ unfamiliarity and are likely to contribute to a supervision need. Also, the age structure of the related patient group further contributes to the above-named issue.

The next finding covers research projects’ preference for custom-built applications rather than repurposing off-the-shelf software. Custom solutions are more expensive but may better fit patient-specific requirements. Commercial software could reduce development costs, but it needs to be open to after-production configuration and tailored to the patient’s needs.

### 4.2. Comparison to Prior Work

Existing reviews in the field, such as those conducted by Choukou et al. [20], Cai et al. [11], and Hill et al. [12], along with other identified publications, discuss the medical effects and clinical outcomes of video game-based interventions in dementia care.

Our review primarily focuses on the technical aspects of applied technologies and development methods in video game interventions for dementia. We analyze the output device and add-on device used in engineering the intervention. Furthermore, we investigate the levels of autonomy and development approaches utilized in these interventions. Through this analysis, we provide insights into practical implementation and technological usage.

Our review finds that most studies still rely on standard computer monitors and custom-designed applications. This suggests a preference for familiar and tailored technologies over advanced or repurposed consumer-grade solutions. Other publications, such as [13], have investigated how studies have been conducted and the acceptance of the technology and the VR user experience. This publication is focused on the applied technology of identified projects and how a clinical trial is conducted in this setting.

### 4.3. Strengths and Limitations

First, the strength of this publication is the time frame, covering the last 20 years of publications. It has been designed to cover all available publications and provide a comprehensive and detailed overview. The second strength is the manual sighting and analysis of the publication, opposing text mining approaches, and the analysis of meta-data. Third, a limitation is that technologies are often not named as technology or products but are described. This does not affect interpretation or outcome, but remembering is essential.

- Exerboard ↔ Balance platform.
- Leap motion ↔ Hand motion tracker.

Fourth, due to funding and resource challenges, only one freely accessible database was used. Using a single source is prone to missing publications. The selected PubMed database is one of the most complete databases in the medical field, and the chance of missing essential publications is considered low but not zero.

Fifth, the publications were analyzed manually, so the possibility of individual bias in the analysis and extraction of the author is unlikely. The chance of an author-induced bias during analysis or extraction is also considered low risk.

Sixth, a lack of diversity in the publication study demographics, as only 10 out of 77 studies are conducted outside of Western countries, limits the applicability of the findings globally.

### 4.4. Future Directions

This study provides a comprehensive overview of the technological landscape in dementia-related video game research. It highlights trends and identifies gaps in the design and usage of the interventions. Its goal is to lay the groundwork for future research and engineers in this emerging field.

This publication is the first of its kind, looking from a medical point of view and detailing the various solutions and technologies applied. It provides a perspective into the last twenty years of research on the cross-section of cognitive impairment and video games. Future iterations can be strengthened by using multiple publication databases and countering bias using more than one screener and extractor.

Using video games to detect and treat patients with cognitive impairments like dementia or Alzheimer’s, its application can benefit medical practitioners, healthcare professionals, and patients alike.

With smaller hardware and more powerful software, upcoming technology will better integrate with existing treatments and workflows and create opportunities for patients, doctors, and healthcare professionals to increase the quality of life for all involved.

Future research should focus on bridging the gap between the use of advanced modern technologies and patient usability and accessibility. By involving caregivers and patients alike in design and research, improved usability can be achieved. Also, it is suggested to investigate autonomous application use with the goal of optimizing healthcare resources and empowering patients to engage in video game activities.

## Figures and Tables

**Table 4:** Description of bibliographic variables.

Variable	Description
**Year of Publication**	Represents the year of the paper’s first publication as stated in the meta-data.
**Country of Origin**	To create geographical context, the country of origin of the publication has been extracted. In the case of multiple authors and affiliations to different organizations, the extracted country was chosen as the first author to be affiliated with the first author.

**Table 5:** Number of publications by country.

Country	Publications	Country	Publications	Country	Publications
United States	14	Germany	5	United Kingdom	2
Netherlands	8	Spain	4	Turkey	2
Canada	7	Greece	4	Italy	2
France	7	Belgium	3	Australia	2
Taiwan	5	Korea	3	Switzerland	2

**Table 6:** Advancement of clinical research.

Type of Clinical Trial	No. of Publications
Single Case Study	2
Feasibility Study	11
Pilot Study	30
Clinical Trial	33

**Table 7:** Output devices used in publications.

Output Device	No. of Publications
Monitor	38
Tablet	20
VR Headset	9
Smartphone	2
Monitor (Touch)	2

**Table 8:** Autonomy and guidance in publications.

Type of Supervision	No. of Publications
Supervised	43
Unknown	19
Unsupervised	13
Mixed	2

**Table 9:** Development regime for dementia care applications.

Development Regime	No. of Publications
Designed	50
Purpose Shift	15
Unknown	12

## Data Availability

Registration and protocol of this review are registered at the OSF (Open Science Framework) with the ID 29azp (https://osf.io/29azp/ (accessed on 10 November 2024)). The full set of extracted data is available via OSF in csv and xlsx formats.

## Abbreviations

The following abbreviations are used in this manuscript:

API	Application Programming Interface
AR	Augmented Reality
DOI	Digital Object Identifier
EEG	Electroencephalography
MCI	Mild Cognitive Impairment
OSF	Open Science Framework
PMID	PubMed Identifier
PRISMA	Preferred Reporting Items for Systematic Reviews and Meta-Analyses
VR	Virtual Reality

## Appendix A. Table of Included Publications

**Table A1:** Table of the included trials, containing the number of subjects, year of publication, and country of publication. The publications are referenced accordingly.

Publication Title	No. of Subjects	Year of Publication	Country of Publication
Gains in cognition through combined cognitive and physical training: the role of training dosage and severity of neurocognitive disorder [16]	322	2015	Greece
The (cost-) effectiveness of exergaming in people living with dementia and their informal caregivers: protocol for a randomized controlled trial [21]	224	2019	Netherlands
The Parkin’Play study: protocol of a phase II randomized controlled trial to assess the effects of a health game on cognition in Parkinson’s disease [22]	222	2016	Netherlands
MIND food and speed of processing training in older adults with low education, the MINDSpeed Alzheimer’s disease prevention pilot trial [23]	180	2019	United States
Can a serious game-based cognitive training attenuate cognitive decline related to Alzheimer’s disease? Protocol for a randomized controlled trial [24]	162	2022	Switzerland
Diagnostic Validity of the Smart Aging Serious Game: An Innovative Tool for Digital Phenotyping of Mild Neurocognitive Disorder [25]	161	2021	Italy
Validation of the Computerized Cognitive Assessment Test: NNCT [26]	147	2022	Spain
COGTIPS: a double-blind randomized active controlled trial protocol to study the effect of home-based, online cognitive training on cognition and brain networks in Parkinson’s disease [27]	140	2019	Netherlands
Effectiveness of home-based and remotely supervised aerobic exercise in Parkinson’s disease: a double-blind, randomised controlled trial [28]	130	2019	Netherlands
Pre-frail older adults show improved cognition with StayFitLonger computerized home–based training: a randomized controlled trial [29]	120	2022	Canada
Physical Training In-Game Metrics for Cognitive Assessment: Evidence from Extended Trials with the Fitforall Exergaming Platform [30]	116	2021	Greece
Exergaming as a Physical Exercise Strategy Reduces Frailty in People with Dementia: A Randomized Controlled Trial [31]	115	2019	Netherlands
The quest for synergy between physical exercise and cognitive stimulation via exergaming in people with dementia: a randomized controlled trial [32]	115	2019	Netherlands
Cost-effectiveness of exergaming compared to regular day-care activities in dementia: Results of a randomised controlled trial in The Netherlands [33]	112	2021	Netherlands
Effects of Exergaming on Cognitive and Social Functioning of People with Dementia: A Randomized Controlled Trial [34]	112	2020	Netherlands
The Aerobic and Cognitive Exercise Study (ACES) for Community-Dwelling Older Adults with or At-Risk for Mild Cognitive Impairment (MCI): Neuropsychological, Neurobiological and Neuroimaging Outcomes of a Randomized Clinical Trial [35]	111	2018	United States
Validation of a Computerized, Game-based Assessment Strategy to Measure Training Effects on Motor-Cognitive Functions in People with Dementia [36]	105	2016	Germany
Effects of virtual reality-based cognitive training in older adults living without and with mild dementia: a pretest–posttest design pilot study [37]	99	2019	Poland
Motor-cognitive effects of a computerized game-based training method in people with dementia: a randomized controlled trial [38]	99	2017	Germany
Efficacy of simultaneous aerobic exercise and cognitive training in subjective cognitive decline: study protocol for randomized controlled trial of the Exergames Study [39]	96	2021	United States
The “Interest Game”: A Ludic Application to Improve Apathy Assessment in Patients with Neurocognitive Disorders [40]	95	2020	France
Efficacy of serious exergames in improving neuropsychiatric symptoms in neurocognitive disorders: Results of the X-TORP cluster randomized trial [41]	91	2021	France
The Smart Aging Platform for Assessing Early Phases of Cognitive Impairment in Patients with Neurodegenerative Diseases [42]	91	2021	Italy
Feasibility of Repeated Assessment of Cognitive Function in Older Adults Using a Wireless, Mobile, Dry-EEG Headset and Tablet-Based Games [43]	89	2021	United Kingdom
A new tool to assess amnestic mild cognitive impairment in Turkish older adults: virtual supermarket (VSM) [44]	89	2019	Turkey
Goalkeeper Game: A New Assessment Tool for Prediction of Gait Performance Under Complex Condition in People with Parkinson’s Disease [45]	74	2020	Brazil
Touchscreen games to detect cognitive impairment in senior adults. A user-interaction pilot study [46]	74	2019	Spain
Effects of exergaming on cognitive functions and loneliness of older adults with cognitive frailty [47]	69	2023	Taiwan
The Effectiveness of a Virtual Reality-Based Intervention on Cognitive Functions in Older Adults with Mild Cognitive Impairment: A Single-Blind, Randomized Controlled Trial [48]	64	2021	Turkey
Episodix: a serious game to detect cognitive impairment in senior adults. A psychometric study [49]	64	2018	Spain
Aerobic and Cognitive Exercise (ACE) Pilot Study for Older Adults: Executive Function Improves with Cognitive Challenge While Exergaming [50]	64	2015	United States
The Effectiveness of a Virtual Reality-Based Tai Chi Exercise on Cognitive and Physical Function in Older Adults with Cognitive Impairment [51]	60	2018	Taiwan
Time course of changes in motor-cognitive exergame performances during task-specific training in patients with dementia: identification and predictors of early training response [18]	56	2018	Germany
Effects of Exergaming-Based Tai Chi on Cognitive Function and Dual-Task Gait Performance in Older Adults with Mild Cognitive Impairment: A Randomized Control Trial [52]	50	2022	Taiwan
Virtual navigation tested on a mobile app is predictive of real-world wayfinding navigation performance [53]	49	2019	United Kingdom
Comparison between a Paper-Pencil Version and Computerized Version for the Realization of a Neuropsychological Test: The Example of the Trail Making Test [54]	48	2019	France
Efficacy of a Web App for Cognitive Training (MeMo) Regarding Cognitive and Behavioral Performance in People with Neurocognitive Disorders: Randomized Controlled Trial [55]	46	2020	France
Kinect Project: People with dementia or mild cognitive impairment learning to play group motion-based games [56]	38	2019	Canada
Evaluation of a novel Serious Game based assessment tool for patients with Alzheimer’s disease [57]	38	2017	Switzerland
Brain Exercising Games with Consumer-Grade Single-Channel Electroencephalogram Neurofeedback: Pre-Post Intervention Study [58]	35	2021	Thailand
Exergaming for people with major neurocognitive disorder: a qualitative study [59]	31	2020	Belgium
Neuropsychological Benefits of Neuro-Exergaming for Older Adults: A Pilot Study of an Interactive Physical and Cognitive Exercise System (iPACES) [60]	30	2017	United States
Smartphone-Based Neuropsychological Assessment in Parkinson’s Disease: Feasibility, Validity, and Contextually Driven Variability in Cognition [61]	27	2021	United States
A Virtual Reality App for Physical and Cognitive Training of Older People with Mild Cognitive Impairment: Mixed Methods Feasibility Study [62]	27	2021	Greece
Computer Activities for Persons with Dementia [63]	27	2015	United States
Cognitive Rehabilitation of Mildly Impaired Alzheimer Disease Patients on Cholinesterase Inhibitors [64]	25	2004	United States
Effects of Home Based Serious Game Training (Brain Talk™) in the Elderly with Mild Cognitive Impairment: Randomized, a Single-Blind, Controlled Trial [65]	24	2023	Korea
The Effects of Exergaming on Executive and Physical Functions in Older Adults with Dementia: Randomized Controlled Trial [17]	24	2023	China
The VITAAL Stepping Exergame Prototype for Older Adults with Major Neurocognitive Disorder: A Usability Study [66]	22	2021	Belgium
Sensor-based balance training with motion feedback in people with mild cognitive impairment [67]	22	2016	United States
“Kitchen and cooking” a serious game for mild cognitive impairment and Alzheimer’s disease: a pilot study [68]	21	2015	France
Design Preferences for a Serious Game–Based Cognitive Assessment of Older Adults in Prison: Thematic Analysis [69]	20	2023	Australia
The Long-term Effects of Immersive Virtual Reality Reminiscence in People with Dementia: Longitudinal Observational Study [70]	20	2022	Taiwan
Physical and Cognitive Stimulation Using an Exergame in Subjects with Normal Aging, Mild and Moderate Cognitive Impairment [71]	18	2016	France
Video game play (Dance Dance Revolution) as a potential exercise therapy in Huntington’s disease: a controlled clinical trial [15]	18	2013	United States
Learning to Detect Cognitive Impairment through Digital Games and Machine Learning Techniques [72]	16	2018	Spain
The Use of Mobile Games to Assess Cognitive Function of Elderly with and without Cognitive Impairment [73]	16	2018	Belgium
Interactive Somatosensory Games in Rehabilitation Training for Older Adults with Mild Cognitive Impairment: Usability Study [74]	15	2022	Taiwan
The interactive Physical and Cognitive Exercise System (iPACES) and trade effects of a 3-month in home pilot clinical trial for mild cognitive [75]	15	2018	United States
Tele-Medicine Based and Self-Administered Interactive Exercise Program (Tele-Exergame) to Improve Cognition in Older Adults with Mild Cognitive Impairment or Dementia: A Feasibility, Acceptability, and Proof-of-Concept Study [76]	14	2022	United States
The Enhanced Interactive Physical and Cognitive Exercise System (iPACESTM v2.0): Pilot Clinical Trial of an In-Home iPad-Based Neuro-Exergame for Mild Cognitive Impairment (MCI) [77]	14	2018	United States
Technikbasiertes Spiel von Tagespflegebesuchern mit und ohne Demenz [78]	14	2016	Germany
Effect of Spatial Disorientation in a Virtual Environment on Gait and Vital Features in Patients with Dementia: Pilot Single-Blind Randomized Control Trial [79]	13	2020	Germany
User Experience of Interactive Technologies for People with Dementia: Comparative Observational Study [80]	12	2020	Portugal
Remote Assessment of Cognitive Impairment Level Based on Serious Mobile Game Performance: An Initial Proof of Concept [81]	12	2019	Korea
Does Practicing with a Virtual Reality Driving Simulator Improve Spatial Cognition in Older Adults? A Pilot Study [82]	11	2020	Canada
Cognitive Training Using Fully Immersive, Enriched Environment Virtual Reality for Patients with Mild Cognitive Impairment and Mild Dementia: Feasibility and Usability Study [83]	11	2020	Korea
A pilot study and brief overview of rehabilitation via virtual environment in patients suffering from dementia [19]	10	2018	Greece
Rehabilitation for dementia using enjoyable video-sports games [84]	9	2010	Japan
Unobtrusive Monitoring of Computer Interactions to Detect Cognitive Status in Elders [8]	9	2004	United States
Seas the day: Co-designing immersive virtual reality exergames with exercise professionals and people living with dementia [85]	7	2021	Canada
A pilot usability study of MINWii, a music therapy game for demented patients [86]	7	2011	France
Participatory design and evaluation of virtual reality games to promote engagement in physical activity for people living with dementia [87]	6	2020	Canada
Tele-Rehabilitation with Virtual Reality [88]	2	2021	Israel
Can people with Alzheimer’s disease improve their day-to-day functioning with a tablet computer? [89]	1	2016	Canada
Two-week virtual reality training for dementia: Single case feasibility study [90]	1	2014	Canada
Development and Co-design of NeuroOrb: A Novel “Serious Gaming” System Targeting Cognitive Impairment in Parkinson’s Disease [91]		2022	Australia

## Appendix B. Extended Search Query

(" dementia "[ MeSH Terms] OR " dementia "[ All Fields] OR " dementias "[ All Fields] OR " dementia s"[ All Fields] OR (" alzheimer disease "[ MeSH Terms] OR (" alzheimer "[ All Fields] AND " disease "[ All Fields ]) OR (" alzheimer disease "[ All Fields] OR (" alzheimer s"[ All Fields] AND " disease "[ All Fields ]) OR " alzheimer s disease "[ All Fields ])) AND (" video games "[ MeSH Terms] OR (" video "[ All Fields] AND " games "[ All Fields ]) OR " video games "[ All Fields] OR (" video games "[ MeSH Terms] OR (" video "[ All Fields] AND " games "[ All Fields ]) OR " video games "[ All Fields] OR (" computer "[ All Fields] AND " games "[ All Fields ]) OR " computer games "[ All Fields]) OR (" exergamers "[ All Fields] OR " exergaming "[ MeSH Terms] OR " exergaming "[ All Fields] OR " exergame "[ All Fields] OR " exergames "[ All Fields ]))

## Appendix C. Exclusion Terms

1 stroke 2 cancer 3 tumor 4 glaucoma 5 bipolar 6 corona 7 covid 8 panic disorder 9 athlete 10 autism 11 eating disorder 12 adhd 13 schizophrenia 14 chronic pain 15 multiple sclerosis 16 traumatic brain injury 17 spinal cord 18 anesthesia 19 robot 20 robotics 21 posttraumatic 22 screening 23 detecting 24 amblyopia 25 alcohol 26 small vessel disease 27 sex 28 cirrho 29 spectroscopy 30 health care provider 31 schizophrenia 32 remission 33 intensive care 34 drug abusers 35 preoperative 36 heart failure 37 phantom limb pain 38 psychosis 39 loss of control eating 40 sway disturbance 41 spastic 42 falls 43 child 44 biochemical 45 borderline 46 phantom 47 hand 48 limb 49 ptsd 50 nanocluster
